# Effects of probiotics on gut microbiota in poultry

**DOI:** 10.3934/microbiol.2025032

**Published:** 2025-09-02

**Authors:** Shanpeng Zhang, Mengjie Yu, Tianqiao Zhao, Yuxuan Geng, Zitong Liu, Xinglin Zhang, Lumin Yu

**Affiliations:** College of Agriculture and Forestry, Linyi University, Linyi, Shandong 276005, China

**Keywords:** probiotics, gut microbiota, poultry, intestinal health, growth performance

## Abstract

Probiotics are living microbes that impart overall health benefits when introduced appropriately. They play important roles in enhancing immunity, inhibiting harmful bacteria, balancing the gut microbiota, and increasing poultry growth performance. In this manuscript, we address the classifications of probiotics, the compositions and functions of the gut microbiota in poultry, and examine the connection between probiotics and the gut microbiota and their roles in promoting the poultry growth. Probiotics are widely used in poultry production, including *Lactobacillus*, *Bacillus*, *Bifidobacterium*, *Streptococcus*, *Enterococcus*, and *Clostridium*, which can exert beneficial effects through various mechanisms, such as increasing the abundance and diversity of the gut microbiota, promoting the secretion of digestive enzymes and antimicrobial substances, optimizing immune microenvironment homeostasis, and enhancing the intestinal barrier. Furthermore, new probiotic products are emerging in poultry production, including prebiotics, synbiotics, and postbiotics. Other novel approaches are used in poultry production to improve their growth and immune performances and inherit beneficial microbial communities, including the integration of probiotics with gut health-promoting agents and the genetic selection of microbiota. The paper demonstrates the potential of probiotics as effective alternatives of antibiotic growth promoters (AGPs) for the promotion of growth performance and intestinal health in poultry production.

## 1. Introduction

Antibiotics are widely used in the global poultry industry to enhance growth performance and prevent diseases [1]. However, their misuse and abuse have caused adverse effects, including the emergence of antimicrobial resistance, the production of antibiotic residues in animal products and ecosystems, the imbalance of the gut microbiota, and the reduction of beneficial bacteria. Studies have shown that the addition of probiotics in animal feed can improve the abundance of beneficial microbiota in the gastrointestinal tract, promote nutrition digestion and absorption, and enhance the host immunity [2]. There is an increased use of probiotics as sustainable alternatives to antibiotic growth promoters (AGPs) in poultry production. This is because probiotics stimulate the intestinal microbiota, which, in turn, can positively modulate the gastrointestinal environment, thereby increasing the abundance of the beneficial bacteria and improving the growth performance and feed efficiency of broilers [3]. Probiotics are defined by the Food and Agriculture Organization (FAO) and the World Health Organization (WHO) as “live microbes which, when administered in adequate quantities, confer health benefits on host organisms” [4]. Probiotics are mostly used in animal husbandry, including *Lactobacillus*, *Bacillus*, *Bifidobacterium*, *Streptococcus*, *Enterococcus*, and *Clostridium* (Table 1), which can exert the beneficial effects through various mechanisms, such as the competitive exclusion of pathogens, the secretion of antimicrobial substances (e.g., bacteriocins, organic acids), the modulation of the host's immunity, and the enhancement of the intestinal barrier [5].

**Table 1.** Probiotics and their influence on animals, and their key benefits.

Microorganisms	Examples	Key Benefits	References
*Lactobacillus*	*Lactobacillus acidophilus*, *Lactobacillus casei*, *Lactobacillu rhamnosus*, *Lactobacillus plantarum*	Regulating host immunity, maintaining microbial balance, enhancing immunity, protecting intestinal barrier, resisting infection, and improving production performance.	[6],[7]
*Bifidobacterium*	*Bifidobacterium longum*, *Bifidobacterium brevis*, *Bifidobacterium pseudocatenulatum*,	Fermenting carbohydrates into short-chain fatty acids, producing bacteriocins, competing with pathogens for nutrients and attachment sites, and promoting intestinal health.	[8]–[11]
*Bacillus*	*Bacillus subtilis*, *Bacillus licheniformis*, *Bacillus velezensis*, *Bacillus coagulans*, *Bacillus amyloliquefaciens*	Resisting heat and acid, producing digestive enzymes and antibacterial substances, increasing villus height, reducing crypt depth, regulating microbiota and increasing serum immunoglobulin, improving nutrient digestion rate, inhibiting intestinal pathogens, improving intestinal health, and promoting chicken growth and immunity.	[12]–[21]
*Streptococcus*	*Streptococcus thermophilus*, *Streptococcus lactis*	Reducing chicken diarrhea, promoting growth, and increasing short-chain fatty acids (acetic acid and lactic acid) in the intestinal tract of broiler chickens.	[2],[22],[23]
*Enterococcus*	*Eterococcus faecium*, *Enterococcus faecalis*	Enhancing digestion, immunity, and intestinal health, promoting mucosal immunoglobulin A, improving weight gain and feed efficiency, and promoting the growth of beneficial lactic acid bacteria.	[24]–[28]
*Clostridium*	*Clostridium butyricum*	producing butyric acid, reducing inflammation, inhibiting pathogens, alleviating oxidative stress, increasing weight gain, improving feed efficiency , and enhancing intestinal barrier.	[29]–[33]

In this review paper, we thoroughly examine the connection between probiotics and the gut microbiota and their roles in promoting poultry growth. By analyzing studies and findings, we will discuss how probiotics and the gut microbiota impact the poultry growth, thus shedding light on approaches and encouraging additional investigations in this rapidly evolving field.

## 2. Probiotics

### 2.1. Lactobacillus

*Lactobacillus* includes *Lactobacillus acidophilus*, *Lactobacillus casei*, *Lactobacillus rhamnosus*, and *Lactobacillus plantarum*, which play a vital role in regulating the gut microbiota, enhancing immunity, maintaining the intestinal barrier, resisting pathogen infections, and improving the production performance [6]. *Lactobacillus* expresses a variety of cell-surface proteins and structural components that adhere to the intestinal mucosa and epithelial cells, and regulates the host's immune response [7].

### 2.2. Bifidobacterium

*Bifidobacterium* is a part of the gut microbiota, which can ferment indigestible carbohydrates through a specialized metabolic pathway, further providing growth substrates for other members of the gut microbiota [8]. *Bifidobacterium* used in poultry production includes *Bifidobacterium longum*, *Bifidobacterium brevis*, and *Bifidobacterium pseudocatenulatum*, which can compete with pathogenic bacteria for nutrients and attachment sites in the gut of chickens, thus helping to maintain the balance of the gut microbiota [9],[10]. Additionally, *Bifidobacterium* ferments arabinoxylan and pectin into short-chain fatty acids (SCFAs) and produces a variety of antimicrobial substances, including lactic acid, acetic acid, bacteriocins, and hydrogen peroxide, thereby inhibiting the growth of pathogenic bacteria [9],[11]. However, further research is needed to fully understand the specific mechanisms of action of *Bifidobacterium* in poultry and optimize their dosage and delivery methods.

### 2.3. Bacillus

As an endophytic spore-forming bacterium, *Bacillus* resists high temperatures and gastric acid and produces various digestive enzymes and antimicrobial substances, which makes it suitable as a feed additive in poultry [12]–[14]. *Bacillus* includes *Bacillus subtilis*, *Bacillus licheniformis*, *Bacillus velezensis*, *Bacillus coagulans*, and *Bacillus amyloliquefaciens*, which can inhibit intestinal pathogens by secreting antimicrobial peptides and secrete highly active proteases, lipases, and amylases to hydrolyze the complex carbohydrates, thus improving the nutrient digestion and absorption [15]–[18]. Villi and crypts are major structures that enhance digestion, absorption, the intestinal barrier, and immunity. A high villus height-to-crypt depth (VH/CD) ratio is a critical determinant to improve the poultry growth efficiency [19]. Previous studies have confirmed that *Bacillus* exhibits an increase in the intestinal villus height, a decrease in the crypt depth, and an increase in serum immunoglobulin (Ig) to enhance the immune response in chickens, thus improving the growth and immunity of chickens [20],[21].

### 2.4. Streptococcus

Some strains of *Streptococcus* play a positive role as probiotics while others are pathogens [22]. *Streptococcus thermophilus* and *Streptococcus lactis* are widely used as probiotics in poultry. Previous studies have shown that the combination of *Streptococcus thermophilus* and *Enterococcus faecium* at specified doses can alleviate diarrhea symptoms in chickens and improve their growth performance [2]. Compound probiotics significantly increase the levels of SCFAs in the intestinal tracts of broilers, with the acetic acid and lactic acid content increasing by 25–30% [23].

### 2.5. Enterococcus

*Enterococcus* is a common bacterium in the intestinal tract of poultry and plays different roles. *Enterococcus* has a positive impact on the host, such as promoting digestion and absorption and enhancing immunity; however, it can also cause diseases under certain conditions, thus affecting the health and production performance of poultry [24]. *Enterococcus faecium* and *Enterococcus faecalis* are commonly found in the intestines of poultry [25]. *Enterococcus faecium* can increase the secretion of IgA in the intestinal mucosa of broilers, thus enhancing the mucosal immune function [26]. Supplementing with *Enterococcus faecalis* can enhance weight gain and the feed conversion rate of broiler chickens [27]. Previous studies have confirmed that *Enterococcus* promotes the growth of beneficial bacteria by increasing the relative abundance of *Lactobacillus* in the intestine, which is beneficial for intestinal health [28].

### 2.6. Clostridium

*Clostridium butyricum* is an important probiotic, and can survive and proliferate in animal intestines [29]. *Clostridium butyricum* ferments dietary fiber to produce butyric acid, which is essential to activate intestinal epithelial cells, maintain colonic homeostasis, inhibit inflammatory and carcinogenic processes, enhance defensive barrier functions, and alleviate oxidative stress [29],[30]. The addition of *Clostridium butyricum* can increase the average daily weight gain (ADG) and decrease the feed-to-gain ratio (F/G) in chickens [31]. Additionally, supplementation with *Clostridium butyricum* can increase the expression of Claudin-1, which is a tight junction protein that plays critical roles in regulating the intestinal epithelial barrier function and preventing macromolecular transmission [32]. A study has found that *Clostridium perfringens* reduces the levels of Claudin-1, but does not affect the expression of Occludin and Claudin-2, while supplementation with *Clostridium butyricum* can increase the expression of Claudin-1 in chickens infected with *Clostridium perfringens*. This finding suggests that *Clostridium butyricum* may mitigate the damage caused by *Clostridium perfringens* to the intestinal barrier [33].

### 2.7. Commercial probiotics

Five commercially effective strains—*Pediococcus acidilactici*, *Enterococcus faecium*, *Bifidobacterium animalis*, *Lactobacillus reuteri*, and *Lactobacillus salivarius*—have demonstrated consistent efficacies against a broad range of common poultry pathogens [34]. The synbiotic PoultryStar®Bro contains three probiotic microorganisms—including *Enterococcus faecium*, *Bifidobacterium animalis*, and *Pediococcus acidilactici*—which significantly reduce lameness in broiler chickens while strengthening their gastrointestinal barrier [35]. Additionally, the supplementation of a commercial *Bacillus*-based probiotic with either *Bacillus subtilis* or *Bacillus licheniformis* between the pullet to lay periods positively influences the feed intake and egg production in Brown pullets and, to some extent, on egg quality [36].

### 2.8. Probiotics regulation

Probiotics predominantly utilized in animal husbandry must be thoroughly characterized and adhere to guidelines established by international organizations such as FAO and WHO. Furthermore, numerous countries have developed their own regulations tailored to the advancement of their poultry industries, with the scope of these regulations significantly varying across nations. Within the European Union, probiotics are authorized as feed additives in accordance with the Community Register of Feed Additives, pursuant to Regulation (European Commission) No. 1831/2003 [34]. This includes strains such as *Bacillus cereus*, *Bacillus licheniformis*, *Bacillus subtilis*, *Enterococcus faecium*, *Pediococcus acidilactici*, *Lactobacillus farciminis*, *Lactobacillus rhamnosus*, *Lactobacillus casei*, *Lactobacillus plantarum*, *Streptococcus infantarius*, and *Saccharomyces cerevisiae* [34]. In the United States, a robust regulatory framework exists for microbial cultures used in food and feed, which is based on the Generally Recognized As Safe (GRAS) notification system [34]. In China, the Regulations on the Administration of Feed and Feed Additives are enforced to oversee the quality and safety of probiotics in feed, thereby outlining specific requirements for probiotic strains and raw materials.

## 3. Gut microbiota

### 3.1. Composition of gut microbiota

**Figure 1.**
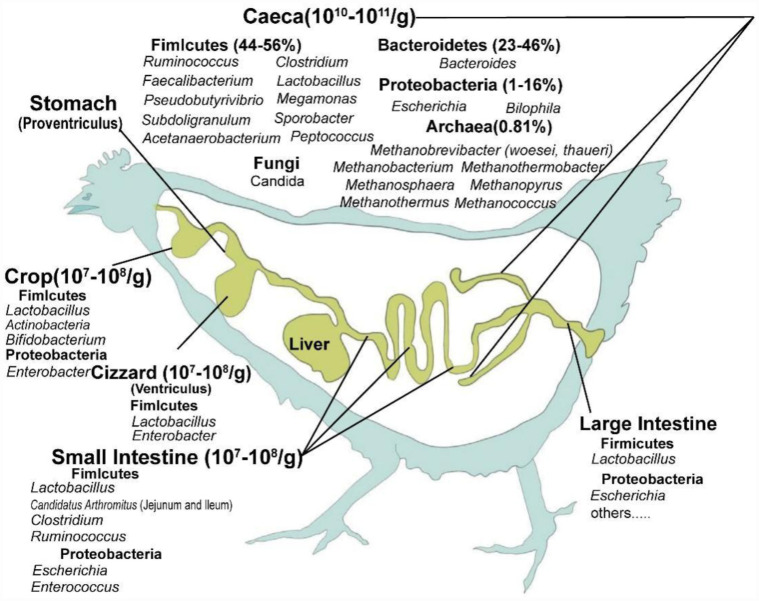
Major taxa surveyed in the chicken gastrointestinal tracts [38]. Only the most common and abundant cecal colonists are listed; viral and phage populations in the chicken gastrointestinal tracts are not presented. Numerous other taxa have been described in the chicken ceca.

The gastrointestinal tracts in poultry are complex organs, including the crop, stomach, gizzard, small intestine, colon, jejunum, caecum, ileum, duodenum, and large intestine, which harbor a wide array of microorganisms (Figure 1). Bacterial phyla have been identified in the intestinal tract of poultry, among which Firmicutes, Bacteroidetes, and Proteobacteria account for over 90% [37],[38]. *Lactobacillus* and *Clostridiaceae* are mainly present in the crop and gizzard [39]. Similar to the duodenum, the chicken ileum is dominated by *Lactobacillus*, which comprises over 68% of its microbiota [40],[41]. *Lactobacillus acidophilus*, *Clostridium*, *Streptococcus*, and *Enterococcus* were found to dominate the ileum in the first three weeks; however, different species of *Lactobacillus* succeed one another as dominant species as the microbiota matures [41].

### 3.2. Functions of gut microbiota

The gut microbiota is an important component of the intestines, representing a highly complex and dynamic ecosystem that plays indispensable roles in the host's physiology, metabolism, and immune homeostasis [42]. The gut microbiota can ferment polysaccharides to produce SCFAs, stimulate the proliferation and differentiation of intestinal epithelial cells, and increase the height of intestinal villi, thereby improving the digestion and absorption capacity of the small intestine and enhancing the feed conversion rate in poultry [43]–[45]. Symbiotic microorganisms hydrolyze polysaccharides, oligosaccharides, and disaccharides into monosaccharides in the cecum, which are further fermented into SCFAs such as acetic acid, propionate, and butyrate [43],[46]–[48]. These SCFAs are mainly absorbed in the cecum and serve as an additional energy source for the poultry [49]. Previous studies have reported that the cecal microbiota expresses a large number of enzymes, including polysaccharide-degrading enzymes and SCFA synthesis enzymes, thus explaining the key role of the cecal microbiota in the host's energy metabolism [46],[50],[51].

### 3.3. Factors affecting gut microbiota

The gut microbiota is generally a dynamic and complex biological system in poultry that plays a key role in physiological functions. A stable, varied, and balanced gut microbiota correlates with the host's health [52],[53]. When the correlation is altered, this induces several metabolic, inflammatory, neurological, and behavioral diseases [54]. The gut microbiota is modulated and shaped by many environmental factors, including diet, circadian rhythm disruptions, and permanent disturbing noises [55]. Additionally, the diversity and composition of the gut microbiota are highly influenced by the acidity of the stomach, the pH of the intestines, the abundance or type of digestive enzymes, and the host's immunity and genome [56]. Therefore, investigating the alterations, diversity, and composition of the gut microbiota will deepen insights into the interactions between the host, environment, and microbes.

## 4. Interaction with probiotics and gut microbiota

Probiotics can promote the growth of the local microbiota via cross-feeding and alleviate intestinal diseases by increasing the abundance and diversity of the gut microbiota, thereby promoting the secretion of digestive enzymes and antimicrobial substances, optimizing immune microenvironment homeostasis, and enhancing the intestinal barrier [57],[58]. Probiotics not only compete with harmful bacteria for the intestinal adhesion sites, nutrients, and growth factors [59], but also inhibit the growth of harmful bacteria by producing organic acids and bacteriocins [60], thereby increasing the abundance of beneficial bacteria. Beneficial bacteria promote gut health by modulating immune responses in the host's gastrointestinal tract [61]. Numerous studies have demonstrated the positive role of probiotics in promoting the production of SCFAs, which participate in bile acid and fat metabolism and signal transduction [62]–[64]. For example, supplementation with a subspecies of *Lactobacillus salivarius* significantly increases the concentrations of propionic acid and butyric acid in the cecum of broilers, and positively modulates the structure of the gut microbiota [65]. The addition of *Bacillus velezensis* Y01 effectively reduces the relative abundance of harmful bacteria in the cecum by positively regulating the structure of the cecum microbial community, promotes the growth performance, and enhances the immune function of Langya chickens [66]. Moreover, three probiotic *Bacillus* strains added to the diets of broilers affect their weights and intestinal morphologies by changing the composition of the gut microbiota [67]. Although the advantages of probiotics are numerous, their cost remains a limiting factor for widespread adoption within the poultry industry [68]. Therefore, it is crucial to explore effective ways to reduce the cost of probiotics while maintaining their quality and effectiveness for wider applications in poultry.

## 5. Future perspectives

Probiotics have been considered viable alternatives to AGPs in animal husbandry; however, the precise mechanisms through which probiotics promote growth remain inadequately understood. The advent of genomics, metagenomics, and metabolomics has significantly enhanced our understanding of the gut microbiota composition and its interactions with the host, thereby enabling the development of precision probiotics tailored to poultry [69]. These precision probiotics will be customized to meet the specific requirements of various poultry breeds, growth phases, and production environments, and are designed to either target particular pathogens or enhance specific metabolic functions within the poultry intestine [70].

Recently, new probiotic products have emerged in the poultry industry, including prebiotics, synbiotics, and postbiotics [71]. Prebiotics are insoluble fibers fermented by specific members of the gut microbiota, including inulin-type fructans, resistant starch, oligosaccharides, β-glucans, and galactooligosaccharides, which are gaining attention in the poultry industry. They play essential roles in improving nutrient digestion and absorption, preventing pathogen adhesion, interacting with the host's immune system, and affecting the intestinal morphology and structure, thereby regulating the intestinal ecosystem and promoting the growth of beneficial bacteria in the gut of poultry [72]. Among these prebiotics, inulin-type fructans and resistant starch improve kidney function, reduce inflammation, and increase the production of SCFAs such as butyrate, which have anti-inflammatory properties and improve the intestinal barrier [73],[74]. Oligosaccharides and β-glucans can improve nutrient digestibility, nitrogen retention, and antibody titers in the ileum [72]. Additionally, galactooligosaccharides enrich Acidobacteriaceae in the cecum and then produce SCFAs such as propionate and valerate, thus inhibiting the growth of *Salmonella* [75]. However, more research is needed to confirm these findings and establish the optimal prebiotic types and dosages for poultry production. Synbiotics, which are a combination of probiotics and prebiotics, synergistically enhance the colonization and activity of beneficial bacteria in the poultry intestine, thus further restoring gut microbiota homeostasis [76],[77]. Postbiotics are metabolites or components of microorganisms, such as enzymes, bacteriocins, exopolysaccharides, and SCFAs, which can offer advantages in terms of stability and safety compared to probiotics [78]. Unlike probiotics, which are live microorganisms, postbiotics are non-living and exert their effects by interacting with the gut microbiota and immune system, thus promoting the host's health [78]. However, further research is needed to fully elucidate the potential of postbiotics and establish the appropriate dosage regimens for poultry production.

Furthermore, the integration of probiotics with gut health-promoting agents, such as phytogenics, organic acids, and digestive enzymes, creates synergistic effects on growth promotion that go beyond the capabilities of individual interventions. The genetic selection of microbiota is a novel approach that helps improve the poultry performance traits and aids in inheriting beneficial microbial communities, thereby significantly enhancing their responsiveness to supplementation with probiotics [79].

Although traditional probiotics used in the poultry industry continue to hold significance, new perspectives are turning these conventional probiotics into precision-targeted functional microorganisms. To advance this field, future research should prioritize the development of cost-effective and high-resolution next-generation sequencing technologies integrated with artificial intelligence (AI)-driven bioinformatics analyses to enhance the identification of functional microorganisms. In addition, real-time monitoring of the gut microbiota dynamics through the use of wearable devices equipped with gut microarrays will facilitate the personalized modulation of probiotics, prebiotics, and postbiotics. This approach will promote the stable colonization of beneficial bacterial strains and ultimately achieve the precise promotion of poultry growth.

## 6. Conclusion

Probiotics have garnered considerable recognition and application in animal husbandry, which is attributed to their safeties and environmentally sustainable characteristics, including being non-toxic, pollution-free, residue-free, susceptible to antibiotics, and lacking virulent determinants and plasmid-linked resistance to clinically relevant antibiotics. However, the future utility of probiotics in the poultry industry demonstrates distinct advantages and challenges compared to other animals. The major reason is in that probiotics contribute to poultry gut health by modulating the gut microbiota, promoting intestinal immune system development, and targeting pathogens such as *Salmonella* and *Clostridium perfringens* through antimicrobial peptides and competitive rejection, thereby directly improving the growth performance, feed conversion efficiency, and carcass quality. The mechanism is similar to their effects on intestinal health and immune regulation in pigs. In contrast, probiotics for ruminant animals, such as cows and sheep, function differently. *Saccharomyces*, a genus of probiotic yeast commonly used in ruminant animals, targets rumen microbiota by enriching cellulose-degrading bacteria and reducing lactate accumulation, thus enhancing fiber digestion and decreasing methane emissions. Collectively, with the advancement of research methodologies and microbiota analysis techniques, personalized selection strategies for probiotic strains may optimize the benefits of probiotics in the contemporary poultry industry, thereby significantly enhancing the growth outcomes of poultry.

## Use of AI tools declaration

The authors declare that they have not used Artificial Intelligence (AI) tools in creating this article.
